# Improving the detection of pathways in genome-wide association studies by combined effects of SNPs from Linkage Disequilibrium blocks

**DOI:** 10.1038/s41598-017-03826-2

**Published:** 2017-06-14

**Authors:** Huiying Zhao, Dale R. Nyholt, Yuanhao Yang, Jihua Wang, Yuedong Yang

**Affiliations:** 1 Institute of Health and Biomedical Innovation, Queensland University of Technology, Brisbane, QLD Australia; 2 Shandong Provincial Key Laboratory of Functional Macromolecular Biophysics, Dezhou University, Dezhou, Shandong China; 3 Institute for Glycomics, Griffith University, Parkland Dr, Southport, QLD Australia

## Abstract

Genome-wide association studies (GWAS) have successfully identified single variants associated with diseases. To increase the power of GWAS, gene-based and pathway-based tests are commonly employed to detect more risk factors. However, the gene- and pathway-based association tests may be biased towards genes or pathways containing a large number of single-nucleotide polymorphisms (SNPs) with small P-values caused by high linkage disequilibrium (LD) correlations. To address such bias, numerous pathway-based methods have been developed. Here we propose a novel method, DGAT-path, to divide all SNPs assigned to genes in each pathway into LD blocks, and to sum the chi-square statistics of LD blocks for assessing the significance of the pathway by permutation tests. The method was proven robust with the type I error rate >1.6 times lower than other methods. Meanwhile, the method displays a higher power and is not biased by the pathway size. The applications to the GWAS summary statistics for schizophrenia and breast cancer indicate that the detected top pathways contain more genes close to associated SNPs than other methods. As a result, the method identified 17 and 12 significant pathways containing 20 and 21 novel associated genes, respectively for two diseases. The method is available online by http://sparks-lab.org/server/DGAT-path.

## Introduction

Genome-wide association studies (GWAS) have successfully identified multiple single nucleotide polymorphisms (SNPs) associated with many diseases. However, most common risk variants can’t be detected for polygenic traits due to weak or moderate effect sizes to account for only a small fraction of the overall phenotypic variation^1^. Consequently, GWAS require enormous sample sizes to identify the majority of risk variants, which remains a considerable challenge for many traits.

Instead of increasing sample sizes, an alternative way is to detect genetic risk factors by aggregating variants over a gene^2–4^ or a cluster of genes^5^. A natural way to define a cluster of genes is the biological pathway that includes a series of actions to produce certain products or changes in a cell. By integrating prior knowledge on genes, pathway analysis is becoming an effective approach to reveal underlying genetic structures of diseases. For example, pathway analysis has been used to detect mechanisms of diseases using both gene expression^6^ and GWAS^7, 8^ data.

Currently, the methods for pathway analysis on GWAS data are mainly divided into two classes according to the type of input data. The first class works with an input of raw genotype data, such as FPCA^9^ and GSEA-SNP^10, 11^. Their applications are limited because raw data is not always available (especially with the shift towards meta-analysis of GWAS summary statistics). In contrast, the other class requires only the association P-value for each GWAS SNP (i.e., GWAS summary statistics)^12–14^. With the relatively high speed and low data requirements, methods using GWAS summary statistics offer the greatest potential. Usually, these methods assess pathway associations through gene associations that are often estimated from P-values of SNPs assigned to the genes via gene-based tests. For example, GSA-SNP represents each gene by the P-value of its k-th best SNP, and evaluates a pathway by calculating a summed Z-score on the P-values of its genes relative to the random chance^13^. KGG^14^ selects the most significant genes according to gene association tests from GATES^2^, and uses the genes to assess the association of pathways through the hyper-geometric test^15^.

One major issue with these approaches is their incomplete adjustment for linkage disequilibrium (LD) correlations between SNPs, where because of the non-random association of alleles at multiple loci due to LD, the statistical associations between alleles at different loci will be different from the expected independent alleles. Therefore, the associations of pathways may bias genes containing a group of significant SNPs with strong LD.

To adjust for LD, many methods have been proposed. For example, ALIGATOR^16^ preselects a list of significantly associated SNPs to avoid LD between SNPs, while it ignores other SNPs. Genomicper^17^ considers the LD relationship within pathways by a special “circular genomic permutation” approach. These methods do not directly use the LD relationship of SNPs in estimating pathway association, and may not completely remove the influence of LD. In contrast, another class of methods explicitly separates SNPs into LD blocks and employs LD blocks for assessment. For example, ProxyGeneLD^18^ divides SNPs in a gene into LD blocks, and adjusts the lowest P-values by the number of LD blocks in the gene. This approach adjusts for LD between SNPs within a gene but ignores the LD between genes in the same pathway. PARIS^12^ separates SNPs in a pathway into LD blocks and counts the number of associated blocks containing at least one significant SNP. The significance of the pathway is then measured by the chance to have a greater number of significant blocks through permutation testing. However, the PARIS uses an arbitrary threshold to define significant SNPs and considers significant blocks equally. Given that an extremely significant SNP should be interpreted differently from a marginally significant SNP, the consideration of all detailed P-values is preferable.

In this study, we developed a new method, DGAT-path, to assess pathways based on detailed P-values of all LD blocks. By selecting the most significant SNP (of the lowest P-value) for each LD block, the p-values are converted into chi-square statistics and summed over all blocks. The summed chi-square score is then compared to those by randomly sampling pathways (permutation), and the chance to have greater scores by the random represents the significance of the pathway. In this way, we avoid the arbitrary threshold used in PARIS, and could consider all LD blocks differently based on detailed P-values. The method, DGAT-path, was found to have lower type I error rate while consistently higher power than other methods. Application to GWAS summary statistics for schizophrenia (SCZ) and breast cancer indicated that DGAT-path detected a higher percentage of associated genes according to the recent GWAS Catalog.

## Methods

### GWAS dataset

In this study, we used meta-analysis summary statistics from genome-wide association studies (GWAS) on the schizophrenia (SCZ) and breast cancer. The SCZ summary statistics were downloaded from the Psychiatric Genomics Consortium (PGC) webpage (http://www.med.unc.edu/pgc/downloads) on 2 June 2014, which includes 1,237,819 SNPs obtained from a schizophrenia GWAS of 9379 cases and 7736 controls^19^. The breast cancer summary statistics were downloaded from the dbGAP with accession number “phs000147.v1.pl”, including 483,123 SNPs obtained from a breast cancer GWAS of 1142 controls and 1145 cases^20^.

### Pathway dataset

A comprehensive pathway dataset was built by collecting the data from KEGG^21^, BioCarta^22^, Gene ontology^23^, REACTOME^24^, and PANTHER^25^ that were downloaded in July 2015. This led to a dataset including 5,764 pathways and 15,718 genes after removing any pathway containing too many (>300) or too few (<5) genes. The pathways were evenly separated into four ranges by the number of involved genes: [5, 9], [10, 16], [17, 36] and [37, 300], where 10, 17, 37 are 25th, 50th, and 75th percentiles of all pathway sizes. Finally, the four ranges include 1747, 1457, 1382, and 1178 pathways, respectively.

### Methods for comparison

We downloaded KGG from http://grass.cgs.hku.hk/limx/kgg/, GSA-SNP from https://sourceforge.net/projects/gsa-snp/, MAGMA (MAGMA-pval-1k version) from https://ctg.cncr.nl/software/magma, PARIS from https://ritchielab.psu.edu/software/paris-download, and Genomicper from https://cran.r-project.org/web/packages/genomicper/index.html. For all methods, the default parameters were utilized in the analysis. We didn’t compare ALIGATOR because it is developed specifically for only the Gene Ontology pathways.

### Pathway analysis

**Figure 1:**
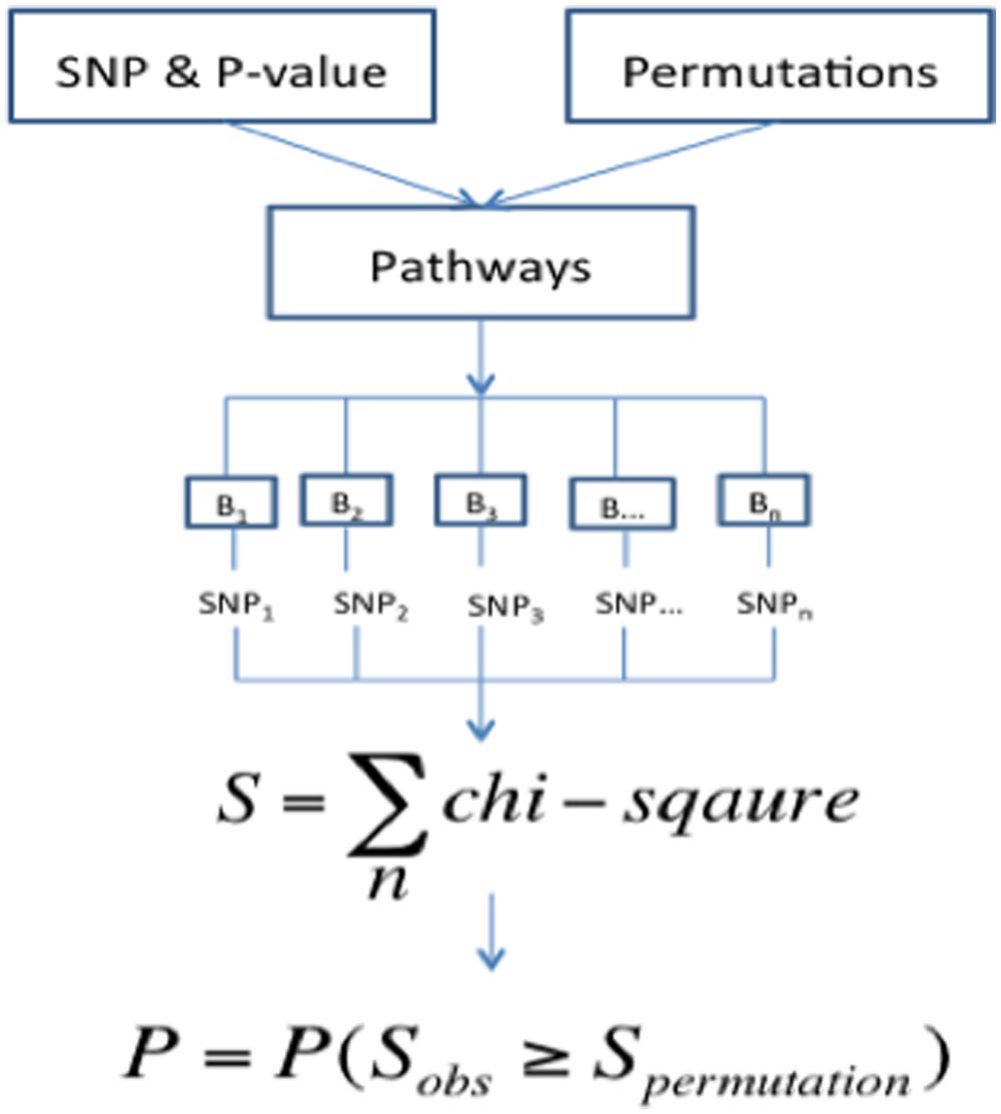
The workflow of DGAT-path to assess pathway associations.

Briefly, as shown in Fig. 1, we first collect all SNPs within genes belonging to a pathway and divide them into blocks according to LD correlations. By representing each LD block with the most significant SNP (of the lowest P-value) inside the block, a score is calculated by summing the chi-square statistics converted from the P-value. The significance of the pathway is defined as the chance to observe the summed chi-square score after randomly sampling pathways (permutation).

Gene sets were obtained from the UCSC genome browser RefSeq (hg19) [downloaded on Mar 20, 2014]. Overlapping isoforms of a gene were combined to form a single full-length version of the gene. For isoforms that didn’t overlap, the isoform containing the most significant SNP (i.e., of the lowest P-value) was kept. This led to 23,438 unique genes. We assigned SNPs to a gene if they mapped to between 15 kb 5′ to the 5′ transcription start site (TSS) and 15 kb 3′ to the 3′ transcription end site (TES). The 15 kb gene boundary extension was chosen because 90% of SNPs affecting expression quantitative trait loci (eQTLs) were observed within this proximity^26^. We employed the package GEC^27^ to divide the SNPs into LD blocks so that the SNPs mapped to a pathway with LD correlations (*r* ^2^) higher than 0.1 were grouped in the same LD block. The pairwise LD correlations were calculated by PLINK^28^ based on 1,000 Genomes Project genotype data (CEU).

The lowest nominal P-value of SNPs in each block was converted to upper-tail chi-square statistics with one degree of freedom, and summed up to provide the observed score (*S*_*obs*_) for each pathway. To calculate a pathway’s significance that is not biased by pathway sizes, we assigned SNPs to pathways, and separated them into LD blocks using the same procedure, from which the same numbers of LD blocks were randomly sampled to calculate permuted scores (*S*_*perm*_). The probability of producing a permuted score larger than the observed pathway score was defined as the association P-value of the pathway. A lower association P-value indicated a more significantly associated pathway. The random sampling was repeated for 1,000 to 1,000,000 times. To speed up the calculations, the sampling will stop when a permuted P-value is >0.05.

In order to directly assess the impact of ignoring LD correlations between pathways, we developed another pathway analysis method, named DGAT-path (gene) that only divided SNPs into LD blocks if the SNPs mapped to the same gene (rather than the same pathway). This method is similar to the KGG method except that DGAT-path (gene) uses a permutation test, whereas KGG uses the hyper-geometric test to assess pathway significance.

Simulation studies for the type I error rate. Simulated pathways were generated by randomly assigning genes to pathways. Through this way, biological relationships were broken between genes while the LD correlations between SNPs were kept. Importantly, this approach needs only GWAS summary statistics, which is different from previous approaches that require original genotype and/or phenotype data to permute phenotype labels or perform genomic randomization. The process was repeated for 1,000 times to generate 1,000 copies of random pathways. By testing each method on the dataset, the percentage of pathways with P-values below 0.05 was calculated as the type I error rate. Additionally, the type I error rates were separately calculated on pathways of four size ranges to measure the impact of the pathway size.

### Power estimation

The power was defined as the proportion of pathways with association P-value below 0.05. For each tested pathway, we multiplied P-values of all SNPs by one factor so that there are a certain percentage of significant SNPs (P-value < 0.05) for the pathway. This is based on a hypothesis that pathways are equally significant if they have exactly the same percentage of significant SNPs. Here, we tested four different percentages of significant SNPs at 30%, 50%, 70%, and 80%. In order to measure the influence of pathway sizes, we have calculated power separately for pathways with different sizes.

## Results

### Simulation studies of type I error rate

**Figure 2:**
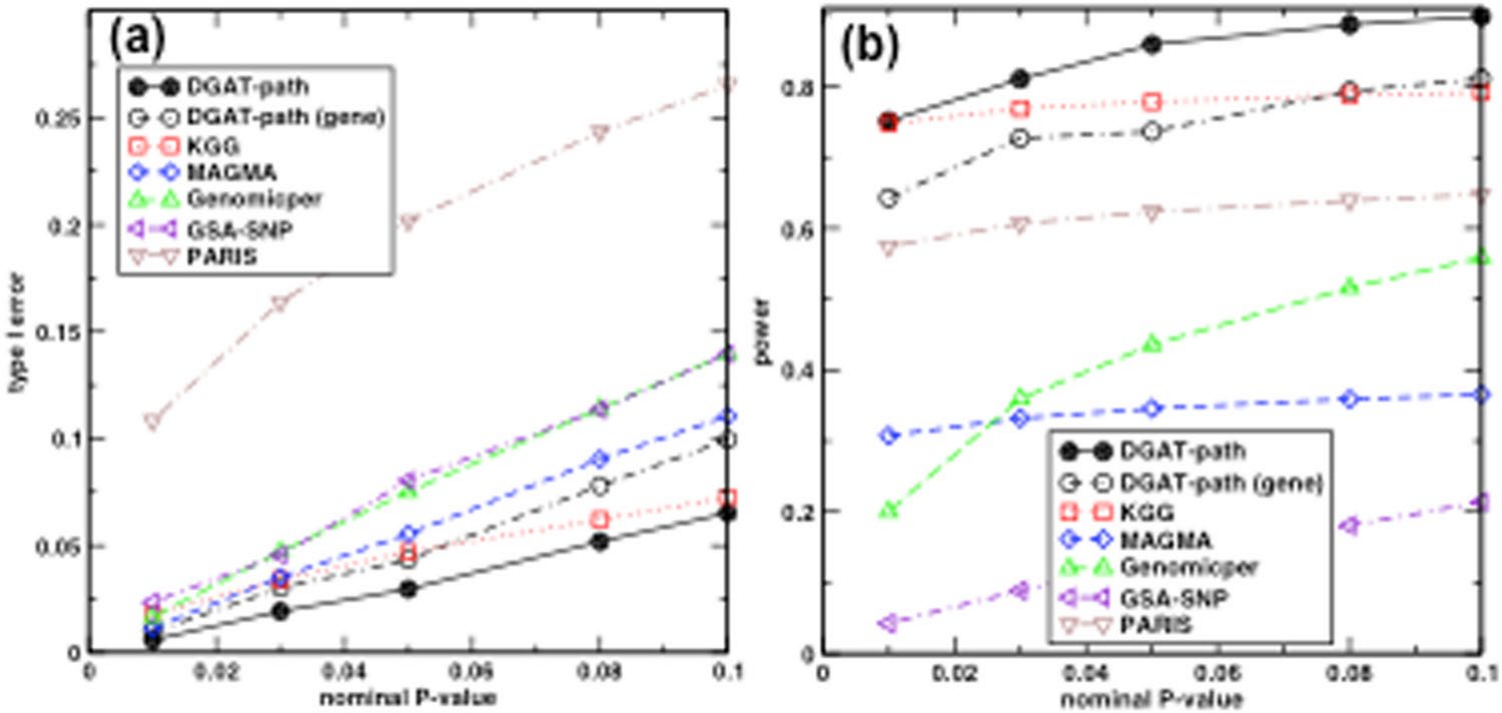
Comparisons of methods by (**a**) the type I error rate and (**b**) the power at different nominal P-values.

As shown in Fig. 2(a), DGAT-path consistently has the lowest type I error rate to detect significant pathways in the random dataset. By comparison, DGAT-path (gene) without considering the LD correlations between genes in pathways constantly increases the error rate by a factor of 50%. KGG has similar performance to the DGAT-path (gene): KGG has a higher type I error rate at low nominal P-value, but lower rate from a nominal P-value of 0.08. These differences are likely caused by different implementation of KGG and DGAT-path (gene). The MAGMA approach shows a slightly but consistently higher error rate than the DGAT-path (gene). GSA-SNP and Genomicper have similar type I error rates that are consistently higher than those of other methods, except for PARIS. While, PARIS shows significantly higher type I error rates that are 1.9 to 4.6 times higher than GSA-SNP.

**Figure 3:**
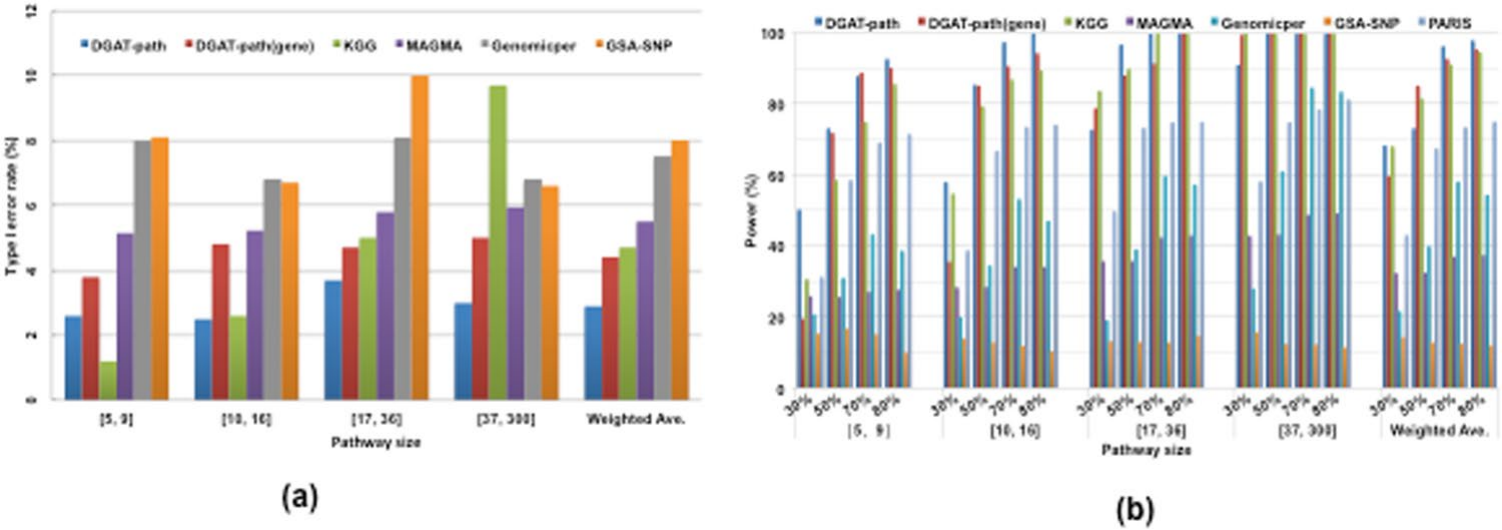
Comparisons of methods under different pathway sizes and percentages of significant SNPs by (**a**) the type I error rates and (**b**) the power at the nominal P-value of 0.05.

To inspect the impact of pathway size, we calculated type I error rates for four different ranges of pathway size. As shown in Fig. 3(a), DGAT-path has a stable type I error rate around 3% for all sizes. DGAT-path (gene) and KGG have similar type I error rates on average (~4.5%), while KGG shows a clear trend of increasing error rates with pathway sizes, which is likely due to an over-estimate of the significance of larger pathways, as large pathways tend to contain more SNPs (genes) in LD. MAGMA has consistently higher type I error rates than DGAT-path (gene). Genomicper and GSA-SNP have similar and higher type I error rates than previous methods. PARIS has significantly higher type I error rates than the other methods for all pathway sizes (Figure S1).

### Power estimation

As shown in Fig. 2(b), DGAT-path outperforms all other methods in power at all nominal P-values. When compared with KGG, although our method is marginally better at a nominal P-value of 0.02, the differences enlarge with an increase in nominal P-value thresholds. At a nominal P-value of 0.1, DGAT-path has 14% higher power than KGG. Similar to the type I error rate, DGAT-path (gene) has a higher power than KGG at low nominal P-values, while a lower power at a nominal P-value > 0.08. PARIS has a stable power of around 0.6 for all four nominal P-values. The power of Genomicper increases from 0.2 to 0.56 when nominal P-values increase from 0.01 to 0.1. MAGMA has a stable power of around 0.35 that is mostly lower than those by Genomicper. GSA-SNP has the lowest power.

Fig. 3(b) shows the power of four methods at different pathway sizes and percentages of significant SNPs (P-value < 0.05). DGAT-path, DGAT-path (gene), and KGG methods show an increasing power with an increased percentage of significant SNPs or pathway size. This is expected given that an increase in the number of significant SNPs will decrease the probability to detect the same effective pathways randomly. In regard to the size of pathways, DGAT-path can clearly detect more pathways of small size than others, which is presumably due to the benefit of dividing pathways into LD blocks and permutation test. In contrast, the DGAT-path (gene) and KGG methods appear to be upwardly biased by significant genes having highly correlated SNP associations. This bias enlarges with an increase in the number of genes within a pathway, and small pathways tend to be downwardly biased. Although two gene-based methods, KGG and DGAT-path (gene), detect more large-sized pathways for SNPs enrichment of 30%, they have a 1.8 and 3 times increase in the type I error rate, respectively. The other four methods have considerably lower power compared to DGAT-path in all cases, and rank as follows from highest to lowest power: PARIS, Genomicper, MAGMA, to GSA-SNP.

### Comparing methods by identification of gene associations reported by GWAS Catalog for the SCZ and breast cancer

The methods cannot be assessed by directly comparing the detected pathways because there is no gold standard to determine truly associated pathways. Such comparison is further complicated given the potential impact of incomplete adjustment for LD correlation between genes mapping to the same pathway. Here, we compared all methods according to the percentage of included associated genes within top detected pathways. A higher percentage of associated genes indicate that the method is more likely to select truly associated pathways. The associated genes were selected as genes closest to SNPs with at least genome-wide *suggestive* association (P < 1 × 10^−5^) within the GWAS catalog database^29^, last updated in year 2016.

**Figure 4:**
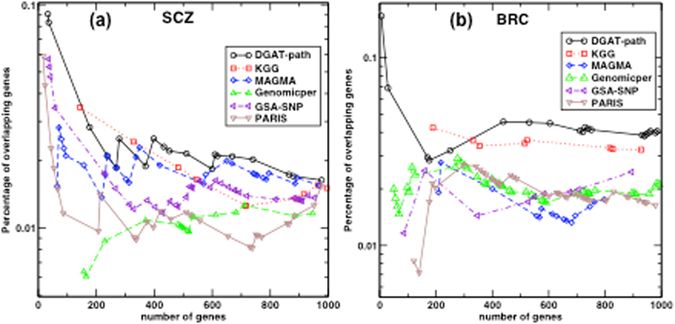
The percentages of genes overlapping with the associated genes from recent GWAS catalogs as a function of the total number of genes in different numbers of top pathways detected by methods on the datasets of (**a**) SCZ (**b**) breast cancer.

Because pathways contain varying numbers of genes, we show the percentage of associated genes versus the total number of genes involved in different pathways. As shown in Fig. 4(a), the DGAT-path generally detected higher percentages of significant genes for SCZ compared to the other pathway methods. For the first two detected pathways, the percentages of DGAT-path are >1.6 times higher than other methods. The difference decreases with an increase in the number of included genes because only a limited number of genes have been detected by GWAS. Hence, we have limited the number of included pathways so that total number of included genes is less than 1000. KGG has all but the 2^nd^ point below the curve of DGAT-path. The sparse points of KGG in the beginning resulted from its bias to pathways of big sizes. Although KGG implicated a higher percentage compared to GSA-SNP for the number of genes below 500, the two methods produce very similar results for larger pathways. MAGMA shows similar, but consistently lower percentages compared to DGAT-path. Genomicper and PARIS generally have the lowest percentages of associated genes.

When applied to the breast cancer data, as shown in Fig. 4(b), DGAT-path performs the best, and has stable ~4% overlapping genes when the top associated pathways contain >400 genes. KGG has close but consistently lower percentages than DGAT-path. The other four methods appear to implicate similar percentages of genes in the GWAS catalog database, which are all lower than the percentages by DGAT-path and KGG.

### Significant associations identified by DGAT-path for the SCZ and breast cancer

By considering multiple test burdens of ~5,000 pathways, the genome-wide threshold for pathway analysis was chosen as 1.1 × 10^−5^ (0.05/5,000). Under this threshold, 17 pathways were identified by our method as significantly associated with SCZ, detailed in Table 1. Among them, “voltage-gated calcium channel activity” is a well-known pathway associated with SCZ^30, 31^. Interestingly, “long-term depression” and “long-term potentiation” were identified significantly associated with SCZ. Indeed, a recent investigation indicated the relationship between SCZ with these two pathways^32^. Moreover, “postsynaptic density”, “ion channel binding pathway”, and “postsynaptic membrane pathway” have been found enriched with genes containing SCZ-related mutations^33, 34^. The “Wnt signaling pathway” has been investigated to detect mutations on genes in SCZ and/or bipolar disorder patients^35, 36^, and the role of this pathway in SCZ has been examined to detect biomarkers^37^. Additionally, 986 genes were included in the significant pathways. Among them, five genes were identified as significantly associated with SCZ by the original study^38^ and 20 novel genes were identified as significantly associated by the recent GWAS study with an expanded sample size^39^.

**Table 1:** Pathways identified by DGAT-path as significantly associated with SCZ and Breast cancer.

Pathway ID	Pathway Name	P-value
SCZ		
GO:0007156	Homophilic cell adhesion	<10^−6^
GO:0007628	Adult walking behavior	<10^−6^
GO:0014069	Postsynaptic density	<10^−6^
GO:0045211	Postsynaptic membrane	<10^−6^
GO:0046415	Urate metabolic process	<10^−6^
path:hsa04713	Circadian entrainment	<10^−6^
path:hsa04724	Glutamatergic synapse	<10^−6^
path:hsa04730	Long-term depression	<10^−6^
GO:0005245	Voltage-gated calcium channel activity	1.9 × 10^−6^
P00019	Endothelin signaling pathway	1.9 × 10^−6^
REACT_18325	Regulation of insulin secretion	1.9 × 10^−6^
GO:0044325	Ion channel binding	3.0 × 10^−6^
path:hsa04270	Vascular smooth muscle contraction	6.9 × 10^−6^
GO:0007169	Transmembrane receptor protein tyrosine kinase signaling pathway	6.9 × 10^−6^
path:hsa04720	Long-term potentiation	6.9 × 10^−6^
P00057	Wnt signaling pathway	7.9 × 10^−6^
path:hsa04261	Adrenergic signaling in cardiomyocytes	8.9 × 10^−6^
Breast Cancer		
GO:0002456	T cell mediated immunity	<9.9. × 10^−7^
GO:0003964	RNA-directed DNA polymerase activity	<9.9 × 10^−7^
GO:0005751	Mitochondrial respiratory chain complex IV	<9.9 × 10^−7^
GO:0007506	Gonadal mesoderm development	<9.9 × 10^−7^
GO:0019031	Yiral envelope	<9.9 × 10^−7^
GO:0042287	MHC protein binding	<9.9 × 10^−7^
GO:0042989	Sequestering of actin monomers	<9.9 × 10^−7^
GO:0050658	RNA transport	<9.9 × 10^−7^
GO:0070544	Histone H3-K36 demethylation	<9.9 × 10^−7^
P00012	Cadherin signaling pathway	<9.9 × 10^−7^
GO:0045211	Postsynaptic membrane	<9.9 × 10^−7^
GO:0045296	Cadherin binding	4.0 × 10^−6^

For the breast cancer GWAS summary statistics, DGAT-path identified 12 pathways with P-value below the threshold (1.1 × 10^−5^) (Table 1). The most significant pathways, “T cell mediated immunity” and “Viral envelope”, have previously been proven to be associated with breast cancer^40–42^. The significant pathways involve 431 candidate genes, among which fourteen genes have been collected by the Cancer Gene Census Category (CGC, http://cancer.sanger.ac.uk/census/) as known breast cancer genes (*CDH11*, *CTNNB1*, *DNM2*, *EGFR*, *ERBB2*, *ERBB3*, *GOPC*, *GRIN2A*, *HNRNPA2B1*, *NDRG1*, *PICALM*, *STRN*, *TCF3*, and *TCF7L2*). Another seven genes (*CDH13*, *CDH7*, *CTNNA2*, *ERBB4*, *GRIK1*, *PCDH15*, and *TCF7L2*) were reported as associated with the breast cancer by recent GWA studies^43–47^.

## Discussion

In this study, we developed a new method, DGAT-path to assess pathway association. Through dividing SNPs in a pathway into LD blocks, a score is calculated by summing chi-square statistics converted from P-values of the most significant SNPs in each LD block. Because the summed chi-square statistics for each pathway are independent, they follow a chi-square distribution with 2*n* degrees of freedom under the null hypothesis (*n* is the number of LD blocks in a pathway). Thus, the summation of the observed independent chi-square statistics reflects the combination of effects of SNPs and provides a valid measure of pathway association. To adjust for pathway size in estimating pathway association, we perform permutations. The method is different from PARIS because PARIS uses a fixed P-value threshold (usually 0.05) to determine significant LD blocks. Additionally, PARIS counts the number of significant LD blocks, thus it considers all significant LD blocks equally, and totally ignores less significant LD blocks. This may explain why PARIS has significantly lower performance than DGAT-path. In addition, PARIS requires setting a parameter to define the significant LD blocks, which may cause low power and high type I error rate when the threshold is not optimized.

The results indicated that the DGAT-path is less affected by pathway sizes with a stable type I error rate ranging from 2.7% to 3.7%. In contrast, the type I error rate of KGG significantly enlarged with an increase in pathway size, most likely because larger pathways have a higher probability to include more correlations between gene association signals. This explanation was supported by the fact that DGAT-path (gene) shows increased type I error rates with an increase in the pathway sizes. Hence, these results support the necessity to adjust for LD correlation among genes in pathway analyses. GSA-SNP appears to appropriately adjust for such potential bias of large pathways via its normalized Z-score by pathway size approach (showing no obvious relationship between the type I error rate and pathway size); however, GSA-SNP did not implicate as many genes according to GWAS catalog data as DGAT-path. This is most likely because GSA-SNP employs the average P-values of a gene set, which increase at the same time. Lastly, although MAGMA produced a lower type I error rate, it detected fewer pathways (had lower power) compared to GSA-SNP. Given that MAGMA was mainly developed for analysis of GWAS raw data, it might not be well optimized for the analysis of GWAS summary statistics.

For the power estimations, the traditional nominal P-value cutoff of 0.05 was initially selected. When a more stringent threshold was utilized to account for testing multiple pathways, i.e., a P-value cutoff of 8.7 × 10^−6^ (0.05/5764), the power of DGAT-path remained the highest among the tested methods, having an average power of 1.4 times higher than KGG and 4 times higher than MAGMA. While GSA-SNP detected very few associated pathways. Genomicper was utilized here by permutation at the SNP level. Genomicper requires a threshold to define the significant SNPs in the permutation test. In this study, we used 0.05 as the threshold. The low performance of Genomicper may result from non-optimized threshold. More details are included in Table S1.

The running times of four methods were tested for pathways of three sizes on an intel i5 CPU of 2.6 GHz, and their runtimes are comparable. As shown in Table S2, GSA-SNP is the fastest and needs eight and fourteen seconds for pathway containing six and 280 genes, respectively. DGAT-path ranks the second, and requires 12 and 34 seconds, respectively. These two methods show an increase of running time with increase of pathway size. By comparison, MAGMA and PARIS have a closely constant running time for three pathway sizes, requiring 50 and 40 seconds, respectively. Since KGG used GUI, its runtimes are not listed. Genomicper (setting the number of permutations to 100,000) took 185, 197, and 2239 seconds for three investigated pathway sizes, respectively.

DGAT-path requires a simple file including chromosome, SNP name, p-value, and location, and a web server has been provided. DGAT-path can be applied to any pre-defined pathway databases, and GWAS summary statistics.
